# Influence of slow oscillation on hippocampal activity and ripples through cortico-hippocampal synaptic interactions, analyzed by a cortical-CA3-CA1 network model

**DOI:** 10.3389/fncom.2013.00003

**Published:** 2013-02-05

**Authors:** Jiannis Taxidis, Kenji Mizuseki, Robert Mason, Markus R. Owen

**Affiliations:** ^1^Centre for Mathematical Biology and Medicine, School of Mathematical Sciences, University of NottinghamNottingham, UK; ^2^Division of Biology, Computation and Neural Systems, California Institute of TechnologyPasadena, CA, USA; ^3^Center for Molecular and Behavioral Neuroscience, Rutgers, The State University of New JerseyNew Jersey, NJ, USA; ^4^Queen's Medical Centre, School of Biomedical Sciences, University of NottinghamNottingham, UK

**Keywords:** hippocampus, slow oscillation, sharp waves, ripples, mossy fibers, temporoammonic pathway, correlations

## Abstract

Hippocampal sharp wave-ripple complexes (SWRs) involve the synchronous discharge of thousands of cells throughout the CA3-CA1-subiculum-entorhinal cortex axis. Their strong transient output affects cortical targets, rendering SWRs a possible means for memory transfer from the hippocampus to the neocortex for long-term storage. Neurophysiological observations of hippocampal activity modulation by the cortical slow oscillation (SO) during deep sleep and anesthesia, and correlations between ripples and UP states, support the role of SWRs in memory consolidation through a cortico-hippocampal feedback loop. We couple a cortical network exhibiting SO with a hippocampal CA3-CA1 computational network model exhibiting SWRs, in order to model such cortico-hippocampal correlations and uncover important parameters and coupling mechanisms controlling them. The cortical oscillatory output entrains the CA3 network via connections representing the mossy fiber input, and the CA1 network via the temporoammonic pathway (TA). The spiking activity in CA3 and CA1 is shown to depend on the excitation-to-inhibition ratio, induced by combining the two hippocampal inputs, with mossy fiber input controlling the UP-state correlation of CA3 population bursts and corresponding SWRs, whereas the temporoammonic input affects the overall CA1 spiking activity. Ripple characteristics and pyramidal spiking participation to SWRs are shaped by the strength of the Schaffer collateral drive. A set of *in vivo* recordings from the rat hippocampus confirms a model-predicted segregation of pyramidal cells into subgroups according to the SO state where they preferentially fire and their response to SWRs. These groups can potentially play distinct functional roles in the replay of spike sequences.

## Introduction

The standard model for memory consolidation assumes that new memories are stored temporarily in hippocampal and parahippocampal areas, and are later transferred to the neocortex, during slow wave sleep (SWS), for long-term storage (Buzsáki, 1989, 2006; Eichenbaum, 2000). However, the question of how information flows between neocortex and hippocampus during SWS is still unresolved. A series of recent observations suggest a general drive from cortex to hippocampus during SWS (Siapas and Wilson, 1998; Sirota et al., 2003; Hahn et al., 2006, 2007; Isomura et al., 2006; Mölle et al., 2006). These studies focus on the temporal relationships between intrinsic rhythmic oscillations found in the cortex and hippocampus, particularly the slow oscillation (SO) and the sharp wave-ripple complexes (SWRs) respectively.

The SO is an intrinsic cortical oscillation (Timofeev and Steriade, 1996; Sanchez-Vives and McCormick, 2000; Timofeev et al., 2000), observed virtually simultaneously throughout all neocortical areas during SWS and anesthesia (Steriade, 2006). It consists of rhythmically recurring (usually <1 Hz) step-like transitions in neuronal membrane potentials between depolarizing envelopes with superimposed action potentials (UP states) and silent, hyperpolarized (DOWN) states. Various theoretical and computational approaches of different architectures and complexities have addressed potential cellular and/or network mechanisms underlying such oscillations (Bazhenov et al., 2002; Compte et al., 2003; Hill and Tononi, 2005; Holcman and Tsodyks, 2006; Parga and Abbott, 2007).

The SWR consists of a large negative local field potential (LFP) peak in the CA1 dendritic layer (sharp wave), accompanied by transient ~150–200 Hz oscillatory patterns (ripples) located in the CA1 pyramidal layer LFP (Buzsáki et al., 1992; Ylinen et al., 1995). Fast-scale replays of temporal patterns of correlated place cells during SWRs (Skaggs and McNaughton, 1996; Lee and Wilson, 2002; Foster and Wilson, 2006; Diba and Buzsáki, 2007) and post-learning memory impairments caused by ripple disruption (Girardeau et al., 2009) indicate a role for SWRs as a possible vehicle for memory transfer to the prefrontal cortical areas (PFC) (Peyrache et al., 2009). SWRs are observed synchronously throughout the hippocampus (Chrobak and Buzsáki, 1996) and are the result of strong depolarizing inputs from CA3 population bursts, exciting CA1 pyramidal cells and interneurons through the Schaffer collaterals (Buzsáki et al., 1992; Csicsvari et al., 2000; Maier et al., 2003). The numerous CA1 interneuron types exhibit diverse spiking responses to SWRs with some types increasing their spiking while others become silent (Klausberger and Somogyi, 2008), potentially serving a mechanism for the replay of the stored place cell temporal patterns (Cutsuridis and Hasselmo, 2010, 2011). Based on the observation that basket cells dramatically increase their firing during SWRs (Klausberger and Somogyi, 2008), firing in phase with ripples (Ylinen et al., 1995; Csicsvari et al., 1999), a recent modeling study of a CA3-CA1 network proposed a ripple generation mechanism, relying on synchronous CA1 perisomatic interneuronal activity and strong fast-decaying recurrent inhibition (Taxidis et al., 2012).

Temporal correlations between neocortical and hippocampal LFPs and unit activity during SO have revealed a strong SO-modulated cortico-hippocampal coupling. The overall cortical activity precedes hippocampal by tens of ms and ripple occurrence increases during UP states (Siapas and Wilson, 1998; Sirota et al., 2003; Isomura et al., 2006; Mölle et al., 2006; Sullivan et al., 2011). In extra- and intra-cellular recordings in the neocortex, entorhinal cortex, and hippocampus of anesthetized and naturally sleeping rodents, the SO was shown to engage entorhinal cells as well, while hippocampal spiking is also SO-modulated, with neurons in the dentate region discharging mostly during cortical UP states (Isomura et al., 2006; Hahn et al., 2007; Sullivan et al., 2011). A study on anesthetized and naturally sleeping rats reported that most CA3 neurons were preferentially active during DOWN states, organized in gamma frequency oscillations, while most CA1 cells fired predominantly in UP states, yielding a correlation of the overall CA1 activity with the SO (Isomura et al., 2006). In contrast, a study on anesthetized mice reported a mixed correlation of CA3 membrane potentials with neocortical UP states, some being depolarized and others hyperpolarized, while most CA1 pyramidal cells were found to be anti-correlated with the SO, being hyperpolarized during UP states (Hahn et al., 2007). Whole-cell recordings from CA1 dendritic R-LM interneurons (Freund and Buzsáki, 1996), also revealed a SO modulation of their membrane potentials (Hahn et al., 2006), indicating that CA1 interneuronal activity can also be SO-modulated.

These results suggest a feedforward control of the SO over hippocampal activity that can potentially be a key feature of memory consolidation processes, with UP states causing neuronal reactivation of newly encoded information in hippocampal circuitry that can be fed back to the cortex during ripples (Mehta, 2007). However, the temporal and functional cross-talk between cortex and hippocampus during sleep and anesthesia and the network mechanisms that control the hippocampal SO-modulation and the UP states-ripples correlation are still not well understood. We try to unravel these mechanisms from a modeling perspective by coupling an established computational model of a cortical network exhibiting SO (Compte et al., 2003) and the aforementioned CA3-CA1 network model reproducing many hippocampal intrinsic dynamics and exhibiting SWRs (Taxidis et al., 2012). The cortical output entrains the hippocampal model via connections to CA3, representing mossy fibers (MF), and to CA1, representing the temporoammonic pathway (TA). We examine how the spiking activity and network dynamics of both CA areas depend on the excitation-to-inhibition balance, induced by the cortical SO output. Our results suggest that the mossy fiber input determines the UP-state correlation or anticorrelation of CA3 population bursts, and consequently SWRs, while the temporoammonic input controls the overall spiking activity of CA1 in relation to UP/DOWN states. Particular ripple characteristics (oscillation frequency, spike-field phase-locking) depend mainly on the Schaffer collateral input to CA1. The combination of both inputs segregates CA1 pyramidal cells into four subsets according to the SO state where they preferentially fire and whether they actively participate in ripple spiking. We verify the model-predicted characteristics of these groups by comparing them against a set of *in vivo* hippocampal recordings from naturally sleeping rats.

## Materials and methods

### Experimental setup, recordings, and *In vivo* data analysis

The experimental data presented here were collected in the lab of György Buzsáki. Detailed information about the surgical, experimental, recording, and spike sorting procedures has been described elsewhere (Csicsvari et al., 1999; Mizuseki et al., 2009, 2011). Briefly, three male Long-Evans rats (250–400 g) were implanted with a 4- or 8-shank silicon probe in the right dorsal hippocampus under isoflurane anesthesia (1–1.5%) and recorded from dorsal CA1 pyramidal layers. Another 4-shank silicon probe was also implanted in the right dorsocaudal medial entorhinal cortex. Each shank had eight recording sites and inter-shank distance was 200 μm. Recordings sites were staggered to provide a two-dimensional arrangement (20 μm vertical separations). The EC probe was positioned so that the different shanks recorded from different layers. The position of the electrodes was confirmed histologically and reported previously in detail (Mizuseki et al., 2009).

LFPs and multiple single neurons activity were recorded from CA1 and EC simultaneously while the animals run on a variety of environments and during sleep sessions, preceding and following the exploration tasks (Mizuseki et al., 2009). Signals were amplified (1000×), bandpass-filtered (1–5 kHz) and acquired continuously at 20 kHz (DataMax system; RC Electronics) at 16-bit resolution. After recording, the signals were down-sampled to 1250 Hz for the LFP analysis. Spike sorting was performed automatically, using KlustaKwik (Harris et al., 2000), followed by manual adjustment of the clusters (Klusters software package, Hazan et al., 2006). Only units with clear refractory periods and well-defined cluster boundaries were included in the analyses (Harris et al., 2000). Classification of pyramidal cells and interneurons of hippocampal and entorhinal cortical neurons was described previously (Mizuseki et al., 2009). All protocols were approved by the Institutional Animal Care and Use Committee of Rutgers University.

SWS periods were detected using the ratio of the power in theta band (6–10 Hz) to delta band (1–4 Hz) of LFP, followed by manual adjustment with the aid of visual inspection of whitened power spectra and the raw traces (Sirota et al., 2008; Mizuseki et al., 2011). DOWN-to-UP transitions were detected by thresholding the smoothed multiunit spiking activity of entorhinal cells. Ripple events were detected by thresholding the bandpassed (140–230 Hz) CA1 pyramidal layer LFP [see Mizuseki et al. (2009, 2011) for details]. The ripple timings refer to the time of each event's spectral peak.

To assess whether CA1 cells fired preferentially during the UP or DOWN states, the firing rate of each cell was calculated for 350 ms around each DOWN-to-UP transition in 100 ms bins, overlapping by 90 ms and was normalized by its total mean. The position of the maximum firing rate point relative to −60 ms from the state transition (where the average firing is at baseline level, see Figure 6A), was used to classify the cell as an “UP-cell” or “DOWN-cell.”

To assess whether each ripple took place during an UP or DOWN state, the closest DOWN-to-UP transition was detected. If the ripple preceded this transition by a time between −60 ms and −350 ms, it was taken to have occurred during a DOWN state (see Figures 6A and C). In all other cases it was assumed to have happened during an UP state. Note that reducing by half the time interval assigned to DOWN-ripples (−60 ms up to −175 ms) reduced the total number of DOWN-ripples but did not severely affect our results.

The peri-ripple firing rate of each cell was calculated for 200 ms around each ripple in 5 ms bins overlapping by 4 ms. The mean firing rate per ripple was calculated for each cell. The baseline firing, used to assess spiking increase during the average ripple, refers to the segments of the mean rate that are more than 40 ms away from the ripple peak. Cells that did not produce any spikes around the DOWN-to-UP or the ripple windows were discarded (2.7%).

All significance tests in section “Correlations of CA1 Pyramidal Spiking to UP-states and Ripples Depend on the Combined Schaffer and Temporoammonic Inputs. Comparison with *In vivo* recordings” refer to *P* = 0.001 2-sample or paired *t*-tests accordingly on the maximum firing rate increase.

### Cortical model

We implemented a computational cortical network model developed in a study of a possible SO-generating mechanism (Compte et al., 2003). In brief, it consists of a one-dimensional array of equidistantly distributed pyramidal cells and interneurons, in a 4:1 ratio. The former are modeled by a two-compartment (dendritic and axosomatic) regular-spiking Hodgkin-Huxley cell, including various ionic currents (Compte et al., 2003), and the latter by the single-compartment fast-spiking Wang–Buzsáki cell (Wang and Buzsáki, 1996). A fixed number of connections between cells is assigned (on average 20 per cell on each cell type), with an implemented heterogeneity among the population. These connections are distributed to the neighbors around each cell with a Gaussian probability distribution, centered on the cell. Excitatory connections are more widespread than inhibitory ones. Excitatory postsynaptic currents (EPSCs) are AMPA- and NMDA-mediated, and inhibitory ones are GABA-mediated. This model was shown to exhibit robust UP and DOWN states, alternating at frequency <1 Hz, closely resembling the cortical SO (Compte et al., 2003, 2008). Spontaneous pyramidal spikes initiate UP states in non-specific sites, which spread throughout the network via recurrent excitation, regulated by inhibition, and are terminated by the buildup of hyperpolarizing currents, particularly the Na^+^-dependent K^+^ current *I*_*KNa*_.

A version of the original model was implemented, including 1000 pyramidal cells and 250 interneurons and a modified synaptic-interaction scheme. Whenever a presynaptic spike is detected (with detection threshold at 10 mV for the membrane potential of the presynaptic cell) a fixed instantaneous increase is given in the postsynaptic cell's synaptic conductance. This increase is set to be 1/3 of the corresponding parameter α_syn_ in Compte et al. (2003) for excitatory connections and 1/2 for inhibitory ones. Moreover, in the original model each synapse in a cell is modeled by an independent post-synaptic variable, whereas in our implementation each cell has one “total” variable for each synaptic type. We therefore adjusted the overall excitation by reducing the original model's maximal excitatory conductances (to 4.15 nS and 0.225 nS for pyramidal-to-pyramidal AMPA and NMDA synapses respectively, and to 0.5 nS and 0.11 nS for pyramidal-to-interneuron synapses. Original values were 5.4, 0.9, 2.25, and 0.5 nS respectively) and we enhanced inhibition by reducing the GABA reversal potential from −70 to −75 mV. Finally, note that the maximum sodium-dependent potassium conductance *g*_*KNa*_, in the dendritic compartment of cortical pyramidal cells has been reduced to 0.5 mS/cm^2^ from the original value 1.33 mS/cm^2^ (Compte et al., 2003). This is done to increase the excitability of pyramidal cells, prolonging (shortening) the duration of UP (DOWN) states. The resulting approximately equal duration of UP and DOWN states allows an unbiased temporal analysis of correlations between hippocampal activity and the two SO states. With these modifications, the network reproduces all the basic characteristic properties of the original one, i.e., alternating UP/DOWN states with frequency <1 Hz (~0.83 Hz) and UP-state firing rates of ~15 Hz for pyramidal cells and ~30 Hz for interneurons.

### CA3-CA1 model

The detailed morphology of the CA3-CA1 hippocampal network model has been extensively described in a previous study of SWRs in the rat hippocampus (Taxidis et al., 2012). Both CA areas consist of one dimensional arrays of 1000 pyramidal cells and 100 interneurons, equidistantly distributed. Pyramidal cells are modeled by the two-compartment (dendritic and axosomatic) Pinsky–Rinzel cell (Pinsky and Rinzel, 1994). All interneurons are considered perisomatic basket cells and are modeled by the Wang–Buzsáki model (Wang and Buzsáki, 1996). Both are Hodgkin–Huxley cell models that accurately capture the firing properties of the respective cell types (Taxidis et al., 2012). All connectivity parameters and synaptic strengths were inspired from the literature so that the final topology of both areas, while simplified, is as realistic as possible [see Taxidis et al. (2012) for details and justification of parameter values]. Briefly, the connectivity scheme is again based on a Gaussian probability distribution of connections around each cell, similar to the cortical model. The SD of the Gaussian is again larger for pyramidal cells than interneurons, mimicking extensive excitatory axons and more locally constrained inhibition (Bernard and Wheal, 1994; Traub et al., 1999).

CA3 is characterized by an extensive recurrent excitatory network, with no inhibition between interneurons. In accordance with anatomical studies (Sik et al., 1993; Traub et al., 1999), pyramidal cells make 55 connections (29% of their total 190) to other CA3 pyramidal cells, five connections (2.6%) to CA3 interneurons and 130 connections (68.4%) forming Schaffer collaterals to CA1 cells. This yields a connectivity probability of 10.62% between any CA3 pyramidal-pyramidal pair [within the estimated 15% upper limit for the intact CA3 (Bernard and Wheal, 1994)] and ~10% connectivity probability for pyramidal-interneuron pairs (Miles et al., 1988; Traub and Miles, 1991). Interneurons make 68 connections on nearby CA3 pyramidal cells, forming multiple contacts with each targeted cell (Traub et al., 1999), resulting in a high connectivity probability within their connections-cluster (Miles et al., 1988; Traub and Miles, 1991).

In contrast to CA3, a strong and dense recurrent inhibitory network underlies CA1 topology, while connections between pyramidal cells are omitted. Interneurons make 400 and 100 connections with nearby pyramidal cells and interneurons respectively, making multiple contacts with each cell (Buhl et al., 1994) and yielding high connectivity probabilities around each interneuron (Bartos et al., 2007). Pyramidal cells contact 20 interneurons on average in their extensive connections-cluster, making mostly monosynaptic connections (Gulyás et al., 1993), yielding a connectivity probability of 30% (Knowles and Schwartzkroin, 1981).

Moreover, CA3 drives CA1 through a set of excitatory connections, depolarizing both pyramidal cells and interneurons, mimicking Schaffer collaterals. Each CA3 pyramidal cell contacts 130 CA1 neurons on average, with connections distributed according to a Gaussian probability centered on the corresponding CA1 pyramidal cell and covering roughly two thirds of the total network length (Li et al., 1994). To include multiple contacts on each targeted cell (Sorra and Harris, 1993) and to implement a strong Schaffer-drive to CA1 (Andersen et al., 2007) 13 synapses are assigned for each Schaffer connection on interneurons. The strength of the Schaffer drive varies throughout the CA1 pyramidal cells, by assigning to each cell a number of synapses per incoming Schaffer connection, taken from a Gaussian distribution: |13 ± 13| (mean and SD), taking the absolute value to avoid negative numbers [see Taxidis et al. (2012) for details]. Thus, a subset of cells receives stronger excitation than average, constituting a “strongly-driven subset.” The numbers of synapses-per-Schaffer connection are changed in some of the simulations to induce an increased Schaffer drive, as explained in the “Results” section.

Both networks have individually been shown to reproduce various anatomical properties and functional features of the respective CA areas. Most importantly, the CA3 network exhibits theta-periodic quasi-synchronized population bursts, with sparse pyramidal spiking, whereas the interneuronal network in CA1 oscillates at gamma frequencies and can synchronize further at ripple frequency range. The full CA3-CA1 model was shown to generate SWRs in CA1 as a response to the CA3 bursts, accurately reproducing all basic ripple characteristics (Taxidis et al., 2012).

### Synaptic interactions and general cortico-hippocampal connectivity

In both the cortical and hippocampal networks all excitatory connections target the dendritic compartment of pyramidal cells, whereas inhibitory ones target the axosomatic compartment. All postsynaptic currents within and between the individual networks are given by:

(1) $$I_{\text{syn}}=\;g_{\text{syn}}s_{\text{syn}}\left(V-V_{\text{syn}}\right)$$

where *g*_syn_ is a fixed conductance, *V* is the membrane potential of the postsynaptic cell and *V*_syn_ is the reversal potential of the synapse. The postsynaptic variable *s*_syn_ is instantaneously increased by a fixed value α_syn_ when an action potential arrives at the synapse and then decays exponentially with decay time τ_syn_ [except for the NMDA currents in the cortical network, as explained in Compte et al. (2003)]. For all relevant parameter values of the individual network and hippocampal models, see Compte et al. (2003) and Taxidis et al. (2012) along with the aforementioned adjustments for the cortical network. All synaptic parameter values, for the cortico-hippocampal connections between the individual models, are summarized in Table 1 and are explained in detail in the following two sections.

**Table 1:** **Parameter values for the instantaneous increase α_syn_ of the postsynaptic variable *s*_syn_ for all types of cortico-hippocampal synapses in the model**.

		**α_syn_**	**EPSP (mV)**	**EPSC (nA)**	**Reported EPSP (mV)**	**Reported EPSC (nA)**	**References**
PFC-CA3	PY	70	5	4.5	2–12	0.2	Yamamoto et al., 1987; Jonas et al., 1993
	IN	0.5	0.25	0.03		0.03	Lawrence et al., 2004
PFC-CA1	PY	2	0.16	0.13		0.16	Otmakhova et al., 2002
	IN	2	1	0.13	1		Empson and Heinemann, 1995

The resulting EPSPs and EPSCs on pyramidal cells (PY) and interneurons (IN) and the ones reported through physiological recordings (where available) are also displayed. All PSPs in the model were measured from a background membrane potential of −74.5 mV for cortical pyramidal cells, −65.3 mV for CA3 pyramidal cells, −62.6 mV for CA1 pyramidal cells and −62 mV for all interneurons.

**Figure 1:**
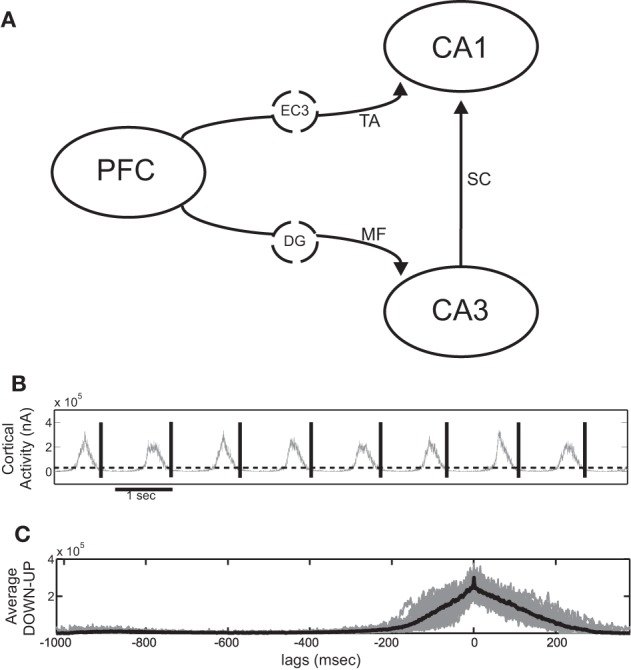
**(A)** Schematic diagram of the full cortico-hippocampal connectivity implemented in our computational network model. The cortex provides input to both CA3 and CA1 through the dentate gyrus (DG) mossy fiber connections (MF) and the entorhinal cortex layer 3 (EC3) temporoammonic pathway (TA). **(B)** Total cortical synaptic activity (grey) of the cortical network during SO. All UP states are detected when the activity exceeds 0.45 × SD and the window between the end of each UP state until the end of the next is taken as an SO cycle (black lines). **(C)** Resulting average SO cycle (black line) of the cortical synaptic activity. (Time 0 ms corresponds to maximum UP-state activity in all figures of average SO cycle).

Figure 1A shows the basic wiring diagram used to couple the cortical and hippocampal model. We add the various coupling mechanisms progressively and examine them individually and in combination. The cortical network is taken to represent the prefrontal cortex (PFC) and sends output to both CA3 and CA1 areas. Since the SO has been shown to extend even to the entorhinal cortex (Isomura et al., 2006; Sullivan et al., 2011), we assume that the cortical output also represents the output produced by the SO of the entorhinal cortex that reaches the hippocampus. CA1 receives direct input from the entorhinal layer 3 (EC3) via the TA, so the PFC-to-CA1 connection represents multistep pathways originating from PFC and reaching CA1 through EC3. The afferent connections in CA3 are the MF originating from the dentate gyrus (DG) which has also been shown to partially participate in the SO (Isomura et al., 2006; Hahn et al., 2007; Sullivan et al., 2011). We therefore assume that the cortical output that is fed into CA3 represents a polysynaptic pathway passing through the entorhinal cortex and the DG. Direct monosynaptic projections from the entorhinal cortex to CA3 are ignored, since their effect is considered to be much weaker than the mossy fiber input (Andersen et al., 2007).

### Connectivity of the mossy fibers and temporoammonic pathway

The direct cortex-to-hippocampus connections in the model represent polysynaptic pathways involving many types of cells and therefore they are highly simplified. Nevertheless, our goal is for both CA areas to receive a realistic input.

MF in the rat hippocampus is mostly oriented transverse to the longitudinal axis of CA3, with very limited overlap in the septotemporal direction (Amaral and Witter, 1989; Andersen et al., 2007). Temporoammonic connections are more widespread in CA1 and are topographically organized, with each EC3 axon covering about one quarter of the septotemporal CA1 axis (Andersen et al., 2007). For simplification, we assign here uniform probability distributions for both types of connections, so that the cortical output will be spread over the entire extent of the CA areas. This is done so that the wave propagation of UP states in the cortical model (Compte et al., 2003) is not reproduced in the hippocampus.

Both MF and the TA have been shown to excite pyramidal cells and interneurons in their respective target areas (Empson and Heinemann, 1995; Acsady et al., 1998; Gabbott et al., 2002), so we implemented connections from cortical cells to both cell types in CA3 and CA1. To reduce further the number of free parameters, we fix the magnitude of the unitary excitatory postsynaptic potentials (EPSPs), so that only the number of connections to pyramidal cells and interneurons varies between different simulations. The EPSP and/or EPSC levels, shown in Table 1, are taken from the literature [partly summarized in Cutsuridis et al. (2010)].

The implemented mossy fiber EPSP to pyramidal cells is 5 mV, within the reported range of 2–12 mV (Yamamoto et al., 1987), although the EPSC is much higher than reported (4.5 vs. 0.2 nA). However, EPSCs up to 1 nA have been recorded (Cutsuridis et al., 2010). The implemented synapse triggers spikes in the CA3 pyramidal cell after a train of four spikes at 40 Hz (not shown) in agreement with recordings (Henze et al., 2002). The implemented EPSC in CA3 interneurons (30 pA) is similar to the recorded quantal EPSC (Lawrence et al., 2004), although a corresponding average EPSP has not been reported.

The TA synapses lie in the stratum lacunosum moleculare of CA1, exciting the distal dendrites of pyramidal cells (Colbert and Levy, 1992). As a result, they are relatively weak and get attenuated along the dendrites, producing very small depolarizations in the somatic compartment that have little effect on the firing properties of the cell (Colbert and Levy, 1992; Empson and Heinemann, 1995; Jarsky et al., 2005). We thus assign a small somatic EPSP magnitude to these connections, 0.16 mV, which is comparable to the Schaffer collateral EPSP (Sayer et al., 1990). A similar magnitude has also been used in a complex simulation of the TA effect on pyramidal cells (Jarsky et al., 2005). The corresponding EPSC peaks at 0.13 nA which is close to the reported 0.16 nA (Otmakhova et al., 2002). Moreover, the TA input exerts a powerful feedforward inhibition in CA1, with EC3 cells possibly targeting strata lacunosum moleculare- and radiatum-lying inhibitory neurons as well (Colbert and Levy, 1992; Empson and Heinemann, 1995). We model this inhibition by assigning direct connections to CA1 interneurons, although our interneurons represent pyramidal-layer perisomatic basket cells. The implemented EPSP is 1 mV, as suggested by recordings (Empson and Heinemann, 1995), and the peak EPSC is the same as in pyramidal cells.

### Simulations of extracellular recordings and detection of UP states, CA3 bursts, and ripples

We model extracellular synaptic activity in the cortex using the total post-synaptic currents from all types of connections, summed over the whole cortical network. All firing rates are calculated by summing spikes from cells of the corresponding network over 30 ms non-overlapping bins.

UP states are detected either (1) through synaptic activity, whenever the total post-synaptic current exceeds a 0.45 × SD detection threshold, or (2) through the average firing rate of all cortical pyramidal cells, calculated in 30 ms non-overlapping bins, whenever it exceeds 0.2 × SD, where SD is the standard deviation of the respective measures over the whole simulation. Any UP states closer than 100 ms are taken as one. By visual inspection, we ensured that both procedures resulted in correct UP-state detection. An SO cycle is considered to begin at the end of a detected UP state and last until the end of the next one, thus including one DOWN state followed by one UP (Figure 1B).

CA3 population bursts are detected through the average firing rate of all CA3 pyramidal cells, calculated in 10 ms non-overlapping bins, whenever it exceeds 1.5 × SD. The bursts' boundaries are set where the firing rate drops below 1.2 × SD. Visual inspection ensured that these criteria detected population bursts correctly for all cases of interneuronal drive by the cortex (*k*_*DG−IN*_, see “Results” section).

Ripples are detected from the 150–200 Hz bandpass filtered total synaptic conductance summed over all connections that would correspond to somatic (pyramidal) layer recordings (so only Schaffer excitatory synapses are ignored). This summation is done over a part of the CA1 network of length 560 μm in the middle of the CA1 array, containing 50 pyramidal cells and six interneurons which we will refer to as the “recording site” throughout the text [see Taxidis et al. (2012) for details and justification]. The root mean square (RMS) of the measure is calculated in bins of 10 ms, overlapping by 50%, and its SD is derived over the whole signal. Ripples are detected when the RMS exceeds a 3 × SD threshold and their boundaries are set where the RMS drops below 2 × SD. Events with less than 20 ms total duration are discarded and neighboring ripples less than 10 ms apart are taken as one event. Similar algorithms were used in Csicsvari et al. (1999, 2000) and Klausberger et al. (2003). Note that spectral analysis of CA1 interneuronal membrane potentials and ripple-related spike histograms include only cells in this “recording site.”

For the averaging over SO cycles, all detected pairs of DOWN-UP states are aligned using the maximum point of the total synaptic current (or firing rate) as reference point (0 ms, Figure 1C), whereas for the averaging over ripples all detected ripple events are aligned over their minimum point. This point is also taken as the reference time of each ripple when aligning them to their corresponding SO state. Finally, when aligning cell spiking with the average SO cycle, the firing rate of each cell is averaged over all cycles and turned into the *z*-score, i.e., its mean is subtracted and it is normalized by its standard deviation.

### Numerical methods

All models were implemented in the Python-based spiking neural networks simulator Brian (Goodman and Brette, 2008), using a second-order Runge–Kutta method for all ordinary differential equations, with a time step of 0.05 ms. Spikes were recorded at every time step, while all other variables were recorded every 1 ms. Data analysis and plotting was performed in MATLAB. Power spectral densities and spike train correlations were computed using algorithms from the Neurospec 20 numerical toolbox (Halliday and Rosenberg, 1999). The CircStat toolbox (Berens, 2009) was used for circular plotting and statistical analysis of pyramidal spike phase-locking to the average ripple. Instantaneous phases of the average ripple were calculated through the Hilbert transform and all phase locking significance *p*-values refer to *V*-tests for non-uniformity of phase distributions with mean direction equal to −90° (corresponding to ripple troughs).

All results presented are taken from simulations producing 40 s of data (except for Figure 7 where 100 s were produced). These contained 32 SO cycles of average duration ~1210 ms (0.83 Hz).

## Results

We study the effects of each implemented cortico-hippocampal connection separately and in combinations, by progressively adding the various contacts between the cortical and the two CA networks. We first explore the role of the mossy fiber input on CA3 population bursts and then the oscillatory responses in CA1 resulting from the Schaffer collaterals. We next add the TA input to CA1 and study its effects on cell spiking, with and without combining it with the Schaffer input. Finally, we compare the predicted correlations with SO states and ripples against a set of *in vivo* recordings, collected in the György Buzsáki lab (Mizuseki et al., 2009, 2011; Sullivan et al., 2011). For clarity, all figures include a diagram with the connections implemented for each simulation.

### Effects of mossy fiber input on CA3

We first examine the effect of the cortical input on CA3 which, when driven by a noisy depolarizing current on all pyramidal cells, exhibits quasi-synchronized population bursts recurring at theta frequencies (Taxidis et al., 2012). Note that the theta rhythmicity is due to the intrinsic bursting frequency range of the CA3 single cell model (Pinsky and Rinzel, 1994).

MF have a very limited spatial extent along the longitudinal CA3 axis, with each granule cell having on average 10–18 large mossy terminals that target only 10–15 different CA3 pyramidal cells (Amaral et al., 1990). To simplify the almost one-to-one mossy fiber projections we assign only one connection from each pyramidal cell in the cortical network (which here represents the DG as well) to CA3 pyramidal cells (*k*_*DG−PY*_ = 1). This connection can reach any cell with uniform probability distribution. Since both networks contain the same number of pyramidal cells (*n* = 1000), each CA3 cell receives on average one input.

**Figure 2:**
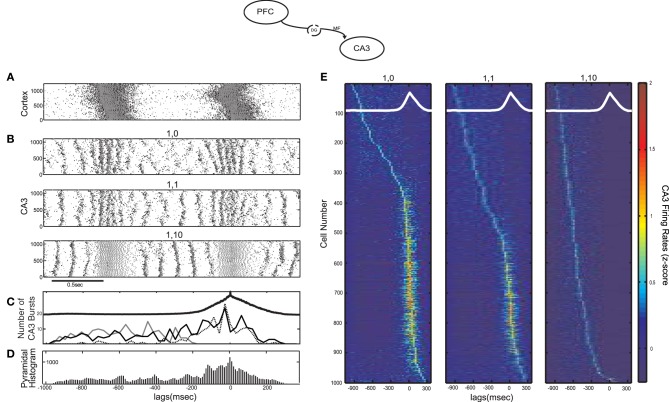
**Influence of the SO on CA3 activity in a computational model of the mossy fiber input on CA3 (model diagram shown on top). (A)** SO in the cortical model acting as input to the CA3 model. Pyramidal cell spikes are shown in black and interneuronal ones in grey (y-axis corresponds to cell numbers in all raster plots). **(B)** Raster plots of the CA3 network when each cortical cell connects to one CA3 pyramidal cell and (from top) 0, 1 and 10 interneurons. The larger the feedforward inhibition in CA3, the less (more) population bursts during the UP (DOWN) state. **(C)** Number of detected CA3 population bursts aligned with the average SO cycle (thick black line) for all three cases of interneuronal drive: 0 interneurons contacted by each cortical cell (dotted line), 1 interneuron (black line) and 10 interneurons (grey line). **(D)** Total spike histogram of CA3 pyramidal cells correlated with the average SO cycle, when each cortical cell connects to one CA3 pyramidal cell and one interneuron. Interneurons exhibit a similar histogram (not shown). **(E)** Color-coded *z*-score normalized average firing rate of each CA3 pyramidal cell (rows), arranged by their peak from top to bottom and aligned with the average SO cycle (white line) calculated from the average firing rate of the whole cortical network (arbitrary units). The three cases of interneuronal input in A are displayed again.

MF have been shown to excite almost 10 times more CA3 interneurons than pyramidal cells, but with much weaker connections involving small *en passant* boutons and very thin (filopodial) extensions of the mossy terminals (Acsady et al., 1998). This can result in an overall feedforward inhibitory input to CA3 from the DG discharges (Mori et al., 2004), although the details of the corresponding circuits are still not well understood. To examine the effect of the feedforward excitation-to-inhibition ratio on the emergence of CA3 bursts we performed simulations with different average numbers of connections from each cortical cell to CA3 interneurons (*k*_*DG−IN*_). Figures 2A,B contain the raster plot of the cortical network exhibiting SO and the resulting CA3 raster plots for three different cases of *k*_*DG−IN*_: no connections to interneurons, one, or 10 connections per cortical cell. There are 1000 cortical pyramidal cells sending output to 100 CA3 interneurons, so, in the latter two cases, each interneuron receives on average 10 × *k*_*DG−IN*_ = 10 and 100 connections respectively.

When the SO output drives only pyramidal cells, (*k*_*DG−IN*_ = 0), the combined strong feedforward and recurrent excitation forces most cells above bursting threshold producing population bursts in UP states, each involving on average ~18% of (non-specific) CA3 pyramidal cells along with their interneuronal targets. The resulting combination of dendritic excitation, somatic inhibition and after hyperpolarization of the bursting cells leaves the network in a disorganized state once the cortical input is removed. At the onset of the DOWN state, population events are less synchronous, involving small cell clusters that produce widespread feedback inhibition, preventing the emergence of population scale bursts. When interneurons receive weak excitation (*k*_*DG−IN*_ = 1), the UP-state bursts become less coherent due to early interneuronal firing desynchronizing pyramidal cells, but DOWN states can now sustain more coherent bursting activity (~10.39% of cells fire on an average burst). In the last case (*k*_*DG−IN*_ = 10), feedforward UP-state inhibition on CA3 pyramidal cells becomes too strong for spikes to occur. In contrast, the increased inhibition and the resulting lack of pyramidal spikes organizes the pyramidal membrane potentials in a more uniform distribution at the onset of the DOWN state, resulting in highly synchronous DOWN-state population bursts (~12.33% of cells fire on an average burst). Figure 2C displays the number of detected CA3 bursts in correlation with the average SO cycle for all three cases of *k*_*DG−IN*_. The two extreme cases of *k*_*DG−IN*_ result in a tradeoff between the CA3 population activity and it's SO-correlation. Strong UP state-driven bursts (*k*_*DG−IN*_ = 0), each involving only parts of the pyramidal population, result in a disorganized DOWN state activity with cells being in various states of excitation, while intense UP-state interneuronal spiking (*k*_*DG−IN*_ = 10) brings pyramidal cells in a uniform (hyperpolarized) state, promoting the ensuing DOWN-state intrinsic CA3 bursting. A similar tendency of interneuronal activity to promote such post-inhibitory “rebound” bursts has been demonstrated through hippocampal electrophysiological recordings (Cobb et al., 1995; Harris et al., 2001; Ellender et al., 2010).

To examine the correlation of individual CA3 pyramidal spiking with the SO for all three simulated cases, the firing rate of each pyramidal cell was calculated and was averaged over the SO cycle, detected through the average firing rate of the cortical network. Figure 2E displays the *z*-score normalized average firing rate of each CA3 pyramidal cell, ordered according to the time of its firing rate peak during the SO cycle. As expected, when *k*_*DG−IN*_ = 0, the majority of cells fire mostly during UP states, whereas when *k*_*DG−IN*_ = 10, pyramidal cells fire almost exclusively during DOWN states. In the intermediate case (*k*_*DG−IN*_ = 1), roughly half the cells fire preferentially during DOWN-state bursts, since they get too inhibited by UP state-induced inhibition to participate in population events, and the rest fire mostly during UP states, where the excitation they receive drives them above spiking threshold. Therefore this connectivity scheme effectively divides the pyramidal population into two distinct groups according to their UP-state response. A similar mixed UP-state correlation and anticorrelation of pyramidal cells was observed in CA3 intracellular recordings on anesthetized mice (Hahn et al., 2007). Moreover by setting each cortical cell to contact one interneuron (*k*_*DG−IN*_ = 1) and one pyramidal cell (*k*_*DG−PY*_ = 1), interneurons receive on average 10 times more connections than pyramidal cells, which is an anatomically realistic ratio (Acsady et al., 1998). We thus keep this regime for the cortico-CA3 connectivity in the rest of this study.

To examine the overall CA3 spiking activity, we detect all SO cycles through the average cortical synaptic activity and superimpose all CA3 pyramidal spikes accordingly to get the total spike histogram of pyramidal cells (Figure 2D). Although roughly half the cells respond preferentially to DOWN states (Figure 2E) the total spiking activity increases during UP states where population bursts are more dense and engage more cells. Interneuronal spiking produces a similar histogram (not shown). We note that intracellular recordings on naturally sleeping rats suggested a preferential CA3 DOWN-state firing (Isomura et al., 2006). Nevertheless, this DOWN-state correlation was associated with gamma-oscillatory population activity (not included in our model), whereas most SWRs (and thus CA3 bursts) were still observed during UP states.

### CA1 responses to Schaffer collateral input

We next examined the characteristics of CA1 responses to CA3 population bursts during SO (with *k*_*DG−PY*_ = *k*_*DG−IN*_ = 1). We omit the TA input, so that CA1 activity is driven purely by the Schaffer collaterals.

**Figure 3:**
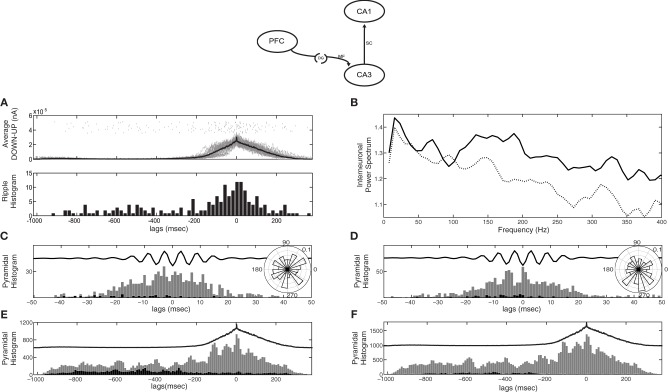
**CA1 spiking activity in the cortico-hippocampal computational model, when the cortical network targets only CA3 (model diagram shown on top). (A)** Raster of detected SWR-like responses (top) and their histogram (bottom) correlated with the average SO cycle (thick line). Most SWRs appear during cortical UP states. **(B)** Power spectral density of the average membrane potential of CA1 interneurons in the default recording site during UP states. With the original Schaffer drive (dotted line), no peak appears at the ripple frequency range (150–200 Hz), unlike in the pure CA3-CA1 network (Taxidis et al., 2012). After increasing the Schaffer collateral-drive, (solid line) a peak at a broad ripple frequency range (100–200 Hz) appears. **(C,D)** Spike histograms of pyramidal cells (strongly-driven subset in grey and moderately-driven in black) correlated with the average ripple-like response (black line, arbitrary units), with low **(C)** and high **(D)** Schaffer drive. Insets depict the spike phase distribution relative to the average ripple. **(E,F)** Spike histogram of CA1 pyramidal cells (strongly-driven subset in grey and moderately-driven in black) correlated with the average SO cycle of the cortical firing rate (black line, arbitrary units), with low **(E)** and high **(F)** Schaffer drive. Spiking of moderately-driven cells decreases during UP states in both cases.

Since CA3 activity depends on the feedforward excitation-to-inhibition ratio induced by the SO, CA1 responses will also depend on it. When the input to CA3 is mostly excitatory (as in the case of *k*_*DG−IN*_ = 1) most CA3 bursts and hence most SWRs are expected to arise during UP states, which agrees with electrophysiological studies (Sirota et al., 2003; Isomura et al., 2006; Mölle et al., 2006; Sullivan et al., 2011). Figure 3A displays the CA1 responses detected through our ripple-detection algorithm (top) and their histogram relative to the average SO cycle (bottom). As expected, more SWR-like events appear during the UP state in our particular setup (*k*_*DG−IN*_ = 1). This picture is reversed when e.g., *k*_*DG−IN*_ = 10 (not shown).

To assess whether these detected events can be considered as ripples, we check if they share the established ripple features that the pure CA1 network has been shown to reproduce (Taxidis et al., 2012). In the isolated CA3-CA1 network, when all CA3 pyramidal cells are driven by a noisy depolarizing current, the average membrane potential power spectral density of CA1 interneurons in the recording site peaks in the range of 150–200 Hz, indicating that interneuronal oscillations underlie ripple generation (Taxidis et al., 2012). The frequency of the spectral peak was shown to depend on the amount of excitation CA1 receives through the Schaffer collaterals, i.e., the more pyramidal spikes in a CA3 burst, the higher the CA1 frequency. In the current cortico-hippocampal model, where the UP states produce both excitation and inhibition to CA3 and there is no input during DOWN states, the drive to CA1 is weaker on average. As a result, the spectral peak is largely lost, even for purely UP-state responses after removing all data segments corresponding to cortical DOWN states (Figure 3B, dotted line), implying that interneurons rarely participate in a synchronous population oscillation. Therefore, the Schaffer input implemented for the pure CA3-CA1 model is, in this case, insufficient to drive interneuronal spiking to ripple frequencies and additional excitatory drive is needed.

As in Taxidis et al. (2012), we split pyramidal cells into two groups: those that receive less than 19.5 synapses-per-Schaffer collateral connection [1.5 × the mean (13) of the Gaussian distribution of synapses per Schaffer input] which constitute ~70% of the population, and those that receive more than 19.5 synapses. We refer to the former group as the “moderately-driven subset” and the latter as the “strongly-driven subset.” Figure 3C shows the total firing histograms of pyramidal cells of both groups, within the CA1 recording site, in correlation with the average bandpassed ripple-like response. Rhythmic spiking in strongly-driven pyramidal cells is not significantly phase-locked to the average signal (*P* = 0.094), with firing peaks not overlapping with ripple troughs (Figure 3C inset). This is an additional indication of the low excitation that CA1 cells receive through the Schaffer collaterals, even during UP-state bursts.

Figure 3E depicts the total spike histograms of pyramidal cells of both groups in correlation with the average SO cycle. Strongly-driven cells (and interneurons, not shown) increase spiking during UP states, where the CA3 bursts involve more cells. In contrast, the majority of pyramidal cells that receive a moderate or weak Schaffer-drive reduce firing during UP states due to increased interneuronal activity. They fire mostly in the DOWN states, when they can overcome the feedforward inhibition. This supports observations on anesthetized mice where CA1 cells were found to be hyperpolarized by UP states (Hahn et al., 2007) but is at odds with recordings on naturally sleeping rats where most CA1 pyramidal cells tended to fire during UP states (Isomura et al., 2006).

Therefore, additional excitation to CA1 is needed to induce UP-state preferential pyramidal spiking and robust ripple activity, i.e., interneurons to fire synchronously at ripple frequencies (150–200 Hz) and strongly-driven pyramidal spiking to be phase locked to ripple troughs. One straightforward way of inducing this additional excitation is by increasing the Schaffer drive to CA1. We thus increased the number of synapses per Schaffer connection in CA1 interneurons to 20. For CA1 pyramidal cells the mean and SD of the Gaussian distribution of synapses per connection was also set to 20 (this increased Schaffer input is implemented in all following simulations). Figure 3B depicts the resulting power spectrum of the average interneuronal membrane potential during UP states (solid line). The peak at ripple frequencies is restored, indicating that the interneurons now receive enough excitation to oscillate synchronously at ripple frequencies. Moreover, the phase locking of the spike histogram of pyramidal cells during the average ripple (Figure 3D) has also been restored, with spikes being significantly locked to the average ripple trough (inset, *P* < 0.001). Nevertheless, the total spike histogram of pyramidal cells during the average SO cycle (Figure 3F) reveals that the majority of cells, those that are moderately-driven by CA3 (black bars), are still inhibited during UP states since they are inhibited during ripples.

Conclusively, a strong Schaffer-drive restores interneuronal ripple oscillations and the strongly-driven pyramidal cell spiking, restoring the main features of ripple events, but does not allow an overall UP-state preferential spiking of pyramidal cells, contradicting reports on naturally sleeping rats (Isomura et al., 2006). Different strengths or distributions of the Schaffer-drive over the pyramidal population, could either make moderately-driven cells fire during ripples, which is inconsistent with the observation of most cells being silent (Ylinen et al., 1995), or it would still prevent them from spiking during ripples, contradicting the *in vivo* UP-state preferential spiking (Isomura et al., 2006). Therefore, an additional, external drive, unrelated to the Schaffer-input is necessary to produce the UP-state spiking, without making the majority of pyramidal cells participate in ripples.

### Effects of Temporoammonic input on CA1

An alternative approach to further increase the drive to CA1 is to assign a direct cortical input through the TA pathway. The importance of the TA for the SO-CA1 coupling has been implied by *in vivo* studies (Dickson et al., 2005; Wolansky et al., 2006) and indirectly by the role of TA input on CA1 spiking and plasticity (Remondes and Schuman, 2002) and on memory consolidation (Remondes and Schuman, 2004).

Although a topographic organization of the EC3 connections to the distal dendrites in the stratum lacunosum moleculare in CA1 has been described (Andersen et al., 2007), the exact details of this circuit are not well understood, e.g., how many CA1 pyramidal cells or interneurons are contacted by a single EC3 axon. The TA input has been shown to have little effect on the firing properties of postsynaptic pyramidal cells, but exerts a powerful inhibitory effect on CA1 (Colbert and Levy, 1992; Empson and Heinemann, 1995). Since inhibition can easily dominate in our network (the fixed pyramidal cell EPSP is much weaker than the interneuronal one, Table 1), we included few connections to interneurons and a larger number to pyramidal cells, so that some can overcome feedforward inhibition.

**Figure 4:**
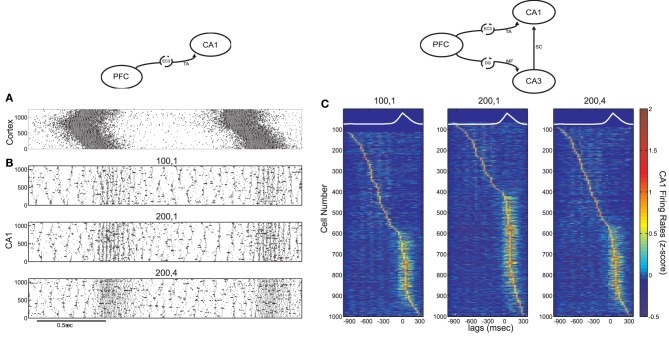
**Influence of the TA input on CA1 activity, in the cortico-hippocampal computational model, without and with Schaffer input (model diagrams are shown on top for each case). (A)** SO in the cortical model acting as input to the CA1 model (pyramidal spikes are in black and interneuronal in grey). **(B)** Three CA1 raster plots with the average number of pyramidal cells and interneurons contacted by each cortical cell, displayed on top. The Schaffer input from CA3 is removed here, to show the effect of solely the TA input. **(C)** Color coded *z*-score normalized average firing rates of CA1 pyramidal cells, arranged by their peak from top to bottom and aligned with the average SO cycle (white line, arbitrary units). The number of cells firing preferentially during UP states depends on the form of the TA input. The increased Schaffer input has been restored here.

To test the isolated TA-input effect on CA1, we first removed the Schaffer input and implemented various values for the numbers of pyramidal and interneuronal cells that each cortical cell targets (*k*_*EC−PY*_ and *k*_*EC−IN*_ respectively). Figure 4A contains a segment of the cortical SO and Figure 4B shows the resulting CA1 spike raster plots with different sets of *k*_*EC−PY*_ and *k*_*EC−IN*_. Note that again an interneuron receives on average 10 × *k*_*EC−IN*_ connections. In all cases, DOWN states are accompanied by sparse spontaneous firing of CA1 pyramidal cells, depolarizing neighboring interneurons. When *k*_*EC−PY*_ = 100 and *k*_*EC−IN*_ = 1, pyramidal activity is similar during UP and DOWN states. Doubling *k*_*EC−PY*_ leads to more pyramidal spikes in the UP states and consequently more interneuronal ones as well, whereas increasing *k*_*EC−IN*_ allows only few pyramidal cells to fire during UP states, with multiple spikes.

**Figure 5:**
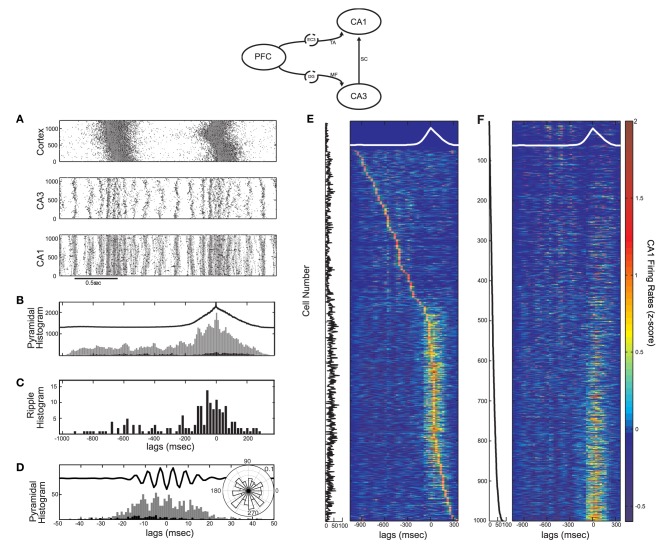
**Activity in the cortico-hippocampal model when the SO drives directly both CA3 and CA1 (model diagram shown on top).** Each cortical cell targets 200 CA1 pyramidal and 2 interneuronal cells in CA1 through the TA input. **(A)** Raster plot of all three networks. **(B,C)** Spike histogram of CA1 pyramidal cells **(B**, strongly-driven subset in grey and moderately-driven in black) and detected ripples histogram **(C)** in correlation with the average SO cycle (black line, arbitrary units). Both pyramidal cell subsets increase spiking during UP states (compare with Figures 3G,H). Ripple occurrence is again correlated with UP states. **(D)** Spike histogram of pyramidal cells in the recording site, aligned with the average ripple, and spike phase distribution relative to the average ripple (inset). The TA input did not significantly affect the spike phase locking (compare with Figure 3D). **(E)** Color coded *z*-score firing rates of CA1 pyramidal cells during the average SO cycle (white line). Cells are stacked according to the peak firing rate, from top to bottom. The number of synapses per Schaffer collateral-connection that each cell receives is plotted on the left. **(F)** Same as **(E)**, for cells stacked according to this number of Schaffer synapses.

After restoring Schaffer collaterals, we calculated the *z*-score normalized firing rates of CA1 pyramidal cells and plotted them, similarly to Figure 2B, for the same combinations of *k*_*EC−PY*_ and *k*_*EC−IN*_ as before (Figure 4C). Cells are again either correlated or anticorrelated with UP states. As expected, increasing only the pyramidal drive results in more UP-state active cells (Figure 4C, middle), while increasing the interneuronal drive, has the opposite effect (Figure 4C, right). Nevertheless, the added TA input is weaker than the Schaffer input, and does not appear to have prominent effects on the features of ripple events. Stronger interneuronal drive (as in the *k*_*EC−IN*_ = 4 case) produces only a small increase of the ripple-frequencies spectral peak of the UP-state average interneuronal potential power spectrum (not shown), while the phase locking of pyramidal spikes to ripple troughs remains significant for all three cases of TA excitation-to-inhibition ratios (see Figure 5D).

Although the TA input does not determine the particular characteristics of the ripple activity, it affects the fraction of pyramidal cells that will overcome UP-state inhibition. Our model suggests that a strong TA-drive to CA1 pyramidal cells aids in reproducing the CA1 UP-state spiking described in Isomura et al. (2006), while its additional drive to interneurons enhances synchronous spiking at ripple frequencies. We thus fix the TA input to *k*_*EC−PY*_ = 200 and *k*_*EC−IN*_ = 2. A segment of the raster plot from all three networks with these parameter values is shown in Figure 5A. The total pyramidal spike histogram (Figure 5B) indicates that the additional TA input lead to the increase of spiking activity of both subsets during UP states (compare with Figures 3E and F). The ripple histogram over the average SO cycle (Figure 5C) and the ripple-correlated spike histogram (Figure 5D) ensure that again most ripples appear during UP states and the phase locking of spikes from the strongly-driven subset to the ripple troughs is still highly significant (*P* < 0.001). Moreover, in this set up, ~11.5% of CA1 cells fire in the average ripple, which is in close agreement with the reported percentage (10%) in naturally sleeping rats (Ylinen et al., 1995).

Finally, to examine the spiking activity of individual CA1 cells, we repeated the *z*-score normalized firing rate calculation for each cell, and arranged them according to either the time of the firing rate peak (Figure 5E) or the number of synapses-per-Schaffer input each cell receives (Figure 5F) which is plotted next to each panel. Figure 5E indicates that, with the implemented combination of TA and Schaffer drive, most CA1 pyramidal cells fire preferentially during UP states [663 cells (70.6%) against 276 (29.4%) firing mostly in DOWN states]. The corresponding Schaffer input is, on average, higher for these cells (the average number of synapses per-Schaffer connection is 28.5 for the “UP-cells” and 13.7 for the “DOWN-cells”). In fact, Figure 5F suggests that the stronger the Schaffer drive, the higher the probability that the cell will fire mostly in UP states (93% of the strongly-driven cells fire mostly in UP states). Note, however, that some cells (7%) with strong Schaffer drive get too inhibited in UP states and tend to fire more in DOWN states, while many of the moderately-driven cells which are silent during DOWN states, fire in UP states due to strong depolarizations from the TA input. In conclusion, the correlation of any particular cell's spiking activity with the SO appears to depend on a combination of both the Schaffer and the TA drive.

### Correlations of CA1 pyramidal spiking to UP-states and Ripples depend on the Combined Schaffer and Temporoammonic inputs. comparison with *In vivo* recordings

Our results suggest that the combination of direct TA- and indirect DG-CA3-input from the cortex can segregate CA1 pyramidal cells into four subgroups, with distinct firing properties related to the SO state where they preferentially fire and the extent of their ripple spiking:

1. Pyramidal cells that are excited by the TA input and receive strong CA3 drive, are expected to fire preferentially during UP states, and participate in all ripples, irrelevant of whether they take place during UP or DOWN states.
2. Pyramidal cells excited by the TA input and receiving average/weak CA3 drive. They should fire preferentially during UP states but their firing increase during any ripple will be minimal.
3. Pyramidal cells inhibited by the TA input, receiving strong/average CA3 drive. They would fire mostly during DOWN states, particularly during the ripples taking place there. UP-state ripples should also transiently increase their spiking activity, since the CA3 input appears to be more critical during the ripple.
4. Pyramidal cells inhibited by the TA input and receiving average/weak CA3 drive. They should fire preferentially during DOWN states and also remain relatively silent during all ripples.

The characteristics of these groups are summarized in Table 2. The model predicts that in the absence of a TA input, subgroups 2 and 3 largely disappear, with the vast majority of cells forming group 4 and a minority in group 1 (Figure 3), as observed in anesthetized mice (Hahn et al., 2007). With the additional TA drive most cells are expected to form groups 2 and 4, with a minority in groups 1 and 3. We tested this segregation on a set of clustered CA1 pyramidal cells (*n* = 2334) recorded from the dorsal CA1 of rats, during SWS preceding or following space exploration tasks (Mizuseki et al., 2009, 2011). Ripples were detected through the CA1 pyramidal layer LFP and DOWN-to-UP transition times refer to entorhinal spiking activity (see section “Materials and Methods”).

**Table 2:** **Summary of the four CA1 pyramidal cell groups suggested by the computational model (see section “Correlations of CA1 Pyramidal Spiking to UP-states and Ripples Depend on the Combined Schaffer and Temporoammonic inputs. Comparison with *in vivo* recordings”), and detected in a set of *in vivo* electrophysiological data**.

	**Strong schaffer collaterals input**	**Average/weak schaffer collaterals input**
Excitatory input from the temporoammonic pathway	Group 1	Group 2
	UP-state preferential firing	UP-state preferential firing
	Large spiking increase during all ripples	Small spiking increase during all ripples
	(17.8%)	(75.8%)
Inhibitory input from the temporoammonic pathway	Group 3	Group 4
	DOWN-state preferential firing	DOWN-state preferential firing
	Large spiking increase during DOWN-state ripples.	Low spiking during any ripple
	Lower spiking increase in UP-state ripples	
	(1.3%)	(5.1%)

They are classified according to their spiking correlation with SO states and ripples. These correlations are associated to the inputs they receive from CA3 and from the entorhinal cortex. Percentages correspond to the ratio of in vivo recorded cells that were found to fall within each category.

**Figure 6:**
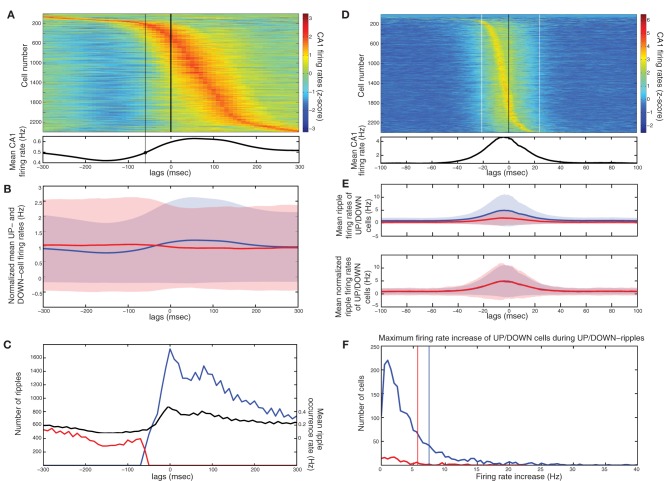
**Analysis on a set of *in vivo* multiunit recordings from the rat CA1 pyramidal layer. (A)** Color coded *z*-score average firing rate of each CA1 pyramidal cell (top) and mean population rate (bottom) around DOWN-to-UP transitions (thick line) assessed by entorhinal neuronal activity. Thin black line indicates the time threshold for segregating cells into UP-state and DOWN-state correlated cells (−60 ms). **(B)** Average firing rates and standard deviations of UP-cells (blue) and DOWN-cells (red), normalized by their total means. **(C)** Number of ripples during UP states (blue) and DOWN states (red), and average ripple occurrence rate (black). **(D)** Color coded *z*-score average firing rate of each CA1 pyramidal cell (top) and mean population rate (bottom) around detected ripple power peaks (black line). White lines indicate the average time limits of detected ripple episodes. **(E)** Mean firing rate of UP-cells (blue) and DOWN-cells (red) around the average ripple (top) and normalized by the mean baseline activity (bottom). **(F)** Histograms of average maximum firing rate increase from baseline of UP cells (blue) and DOWN-cells (red) during UP- and DOWN-ripples respectively. Lines indicate the 1.5 × mean increase, for the classification into highly- and low-firing cells.

In accordance with (Isomura et al., 2006) average spiking of cells around DOWN-UP transitions was typically correlated with either the pre-transition or the post-transition segment (Figure 6A) and all cells were split into two groups (referred to as “UP-cells” and “DOWN-cells”) according to the SO state they correlate with (see “Materials and Methods”). 93.6% (*n* = 2185) were classified as UP-cells and 6.4% (*n* = 149) as DOWN-cells (Figure 6B). According to our model, this implies that the TA input acts as an excitatory drive to the majority of CA1 neurons during SWS, promoting the emergence of groups 1 and 2.

All recorded ripple episodes were also separated into events that took place during a DOWN state (referred to as DOWN-ripples) or an UP state (UP-ripples). 90.1% of events (*n* = 111214) were classified as UP-ripples and 9.9% of events (*n* = 12229) as DOWN-ripples (Figure 6C). Note that the average occurrence rate of ripple episodes is much higher in UP states, therefore the large abundance of UP-cells may also be related to the increased number of SWRs during those (apart from the TA input).

We examined the correlation of individual cell spiking with ripples, where CA3 is expected to provide the prominent drive. Most cells increased their firing around the average ripple with the majority of cells exhibiting a firing peak 3–6 ms before the ripple peak [Figure 6D, in accordance with (Csicsvari et al., 1999)]. Figure 6E depicts the mean firing rate of UP- and DOWN-cells around the average ripple. UP cells show a significantly higher rate increase (*P* < 0.001). This may be due to a stronger input from CA3 (more coherent and/or widespread CA3 population busts in UP states) and/or the additional TA input. When normalizing the average firing rates by the mean baseline activity around the ripples, the relative spiking increase becomes equal for both cell groups, implying that the Schaffer-input received by either group provides a similar relative gain. Therefore, as in our model, the TA input seems unlikely to severely affect ripple spiking responses. We return to this issue further below.

**Figure 7:**
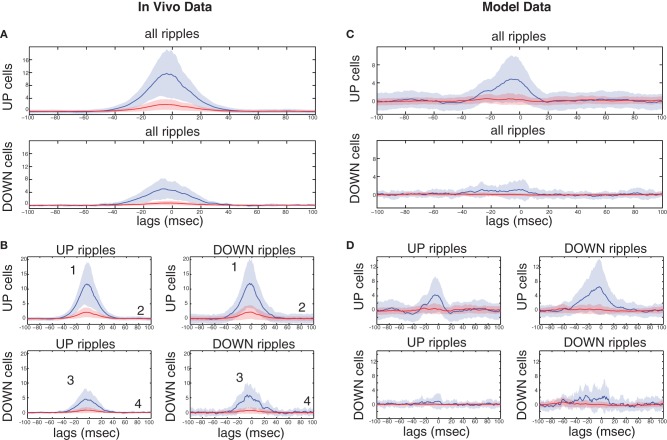
**Ripple-correlated spiking of highly- and low-firing UP- and DOWN-cells in electrophysiological recordings during natural sleep and in the model. (A)** Top, average firing rate increase (Hz) from baseline of highly-firing (blue) and low-firing (red) UP-cells during ripples. Bottom, same for DOWN-cells. **(B)** Average firing rate increase (Hz) from baseline of highly-firing (blue) and low-firing (red) UP- and DOWN-cells during UP- and DOWN-ripples. Numbers indicate which of the cell groups, summarized in Table 2, each line correspond to. **(C,D)** Same as **(A)** and **(B)** respectively, computed on a set of 100 s-long data produced by a simulation of our model. The model captures in a qualitative fashion, the SO-related ripple-spiking characteristics of the various cell subgroups.

Next, each (UP-) DOWN-cell was classified according to its average firing rate increase during (UP-) DOWN-ripples. The classification threshold was set to 1.5× the mean firing rate increase of (UP-) DOWN-cells during the average (UP-) DOWN-ripple (similarly to the model where strongly-driven cells were set by a 1.5 × the mean synapses-per-Schaffer input threshold). 19% of UP-cells (*n* = 415) and 20.8% of DOWN cells (*n* = 31) were classified as “highly-firing” (Figure 6E). Together, these cells constitute 19.1% of the total population, close to the percentage of strongly-driven cells in our model (30%). Figure 7A depicts the average firing rate increase of both subsets of UP-cells (top) and DOWN-cells (bottom) around the average ripple. Cells classified as highly-firing show significantly higher rate increases during ripple episodes (*P* < 0.001 for UP-cells and DOWN-cells). As in Figure 6E, the depolarizing inputs received during UP states induce a significantly larger spiking increase to the two UP-cell groups than the corresponding DOWN-cells (*P* < 0.001 for both highly-spiking cells and weakly-spiking cells). This implies that either DOWN-cell receive weaker inputs from CA3 or that the TA input aids UP-cell spiking during ripples.

To clarify this further, we compared the spiking increase of all four groups of cells during the ripples of their corresponding SO state and those of the opposite state (Figure 7B). UP-cells with either strong or weak spiking during UP-ripples (i.e., those that fall into groups 1 and 2 in Table 2), exhibit a similar increase in DOWN-ripples (*P* = 0.305 and 0.031 respectively). DOWN-cells that fall into groups 3 and 4 also show similar maximum firing increases in UP-ripples and DOWN-ripples (*P* = 0.133 and 0.32 respectively). These results support that different sets of CA1 neurons receive Schaffer-drive of different intensity during SWRs, but the drive to each set is roughly fixed, irrelevant of the SO state where the event took place. Moreover, the TA input, either excitatory or inhibitory (for UP- and DOWN-cells respectively) does not affect the firing rate of cells during the ripple duration.

Figures 7C,D correspond to the same measures calculated for the model data. In the model, the strongly-driven DOWN-cells (segregated as before, based on the synapses-per-Schaffer input) show a particularly lower ripple-spiking than the corresponding UP-cells. Note that the average number of synapses-per-Schaffer input that the former receive is 37.5 vs. 43.6 for the latter. Therefore, the model suggests that the reduced firing of DOWN-cells during ripples is due to a weaker CA3-input. The differences between spiking during UP- and DOWN-ripples are again non-significant in all cell groups (Figure 7D). The only exception being cells in group 1 that increase their firing rate significantly more in DOWN-ripples (*P* < 0.001), since firing rates outside UP-ripples are high (unlike in the data where UP and DOWN activity in CA1 is not as distinct as in the model). Therefore, the TA input again appears not to influence spiking during the actual ripples, whereas the CA3-drive is the critical factor. Conclusively, cells that receive the highest input from CA3 will fire preferentially in UP states where SWRs are more frequent. Those that receive a weaker drive can still fire in ripples, but the TA input can either shift them to an UP-state or DOWN-state correlation. Cells with weak CA3 input will depend on the TA for their correlation with the SO and will not actively participate in ripple spiking. Finally, note that the reduced spiking of DOWN-cells during UP-ripples in the model is mainly due to the TA inhibition that appears somewhat stronger in our model than what is suggested by the recordings.

## Discussion

Various studies have addressed the impact of the cortical SO on other brain areas including the thalamus and hippocampus, focusing on correlations between their intrinsic rhythms and the SO states (Siapas and Wilson, 1998; Sirota et al., 2003; Battaglia et al., 2004; Isomura et al., 2006; Mölle et al., 2006). These studies support the fundamental role of the SO in coupling rhythmical activity of thalamic and hippocampal circuits, with the intense cortical UP-state spiking exerting a strong drive on both areas, promoting their oscillatory events. The spiking activity of the various hippocampal areas during the SO has also been recently studied (Isomura et al., 2006; Hahn et al., 2007; Sullivan et al., 2011), occasionally with contradictory results.

To suggest potential functional mechanisms giving rise to the observed UP state-hippocampal activity relationships and to the contradictions between observations under different experimental protocols, we coupled a SO-exhibiting cortical network (Compte et al., 2003, 2008) with a CA3-CA1 network model of SWRs (Taxidis et al., 2012). The implemented cortico-hippocampal connectivity (Figure 1) is a simplification of the direct and indirect projections between PFC and hippocampus. We focused our analysis on how the spiking activity and intrinsic network dynamics of the two CA areas depend on the excitation-to-inhibition balance, induced by the cortical SO. To our knowledge, this is the first modeling study of the SO effects on hippocampal spiking and ripples. The main results from our simulations can be summarized as follows: (1) The correlation of CA3 population bursts, and corresponding CA1 SWRs, with the cortical SO are controlled by the feedforward excitation-to-inhibition ratio induced by the mossy fiber input. Pyramidal-targeted inputs on CA3 lead to SWRs correlated with UP states, whereas an anticorrelation exists when feedforward inhibition is promoted by the mossy input. (2) Ripple characteristics (interneuronal oscillation frequency and timing of pyramidal spikes) are controlled by the Schaffer input to CA1. This varies between CA1 cells but remains constant between UP-state and DOWN-state ripples. (3) The overall amount of CA1 spiking in relation to UP/DOWN states is affected by the excitation-to-inhibition ratio induced via the temporoammonic TA input. Stronger drive on CA1 pyramidal cells, than on interneurons, promotes UP state-correlated CA1 pyramidal spiking and vice versa, without affecting ripple characteristics. (4) The combination of CA3 and TA inputs on CA1 segregates pyramidal cells into different groups depending on the SO state where they preferentially fire and whether they substantially spike during ripples or not. These groups, confirmed by *in vivo* recordings, may have distinct functional roles during SWS and the ensuing memory consolidation.

These observations are in agreement with previous Granger causality-based (Baccalá and Sameshima, 2001) statistical analysis, reporting that the mPFC drives hippocampal activity particularly during short cortical UP-like states in anesthetized rats (Taxidis et al., 2010).

### Mossy fiber input

Our model-suggested tradeoff between CA3 population activities during the two SO states is generated by the CA3 network's intrinsic dynamics. While pyramidal cells receive the UP-state input, weak or no drive to interneurons allows the network to self-organize into the population bursts seen in the isolated CA3 network (Taxidis et al., 2012), but leaves it in a disorganized state during DOWN states, where cells are in various levels of excitation, leading to poor DOWN activity. In contrast, when interneuronal spiking dominates UP-states, pyramidal cells are left in a more uniform hyperpolarized state at the onset of DOWN states, where they can easily synchronize in spontaneous population bursts. Such a tendency of interneuronal activity to synchronize post-inhibitory pyramidal spiking has also been demonstrated in CA3 and CA1 in *in vitro* slices (Cobb et al., 1995; Ellender et al., 2010) and *in vivo* recordings (Harris et al., 2001). Since these bursts will produce SWRs in CA1, the SO-induced excitation-to-inhibition ratio in CA3 will determine whether ripple occurrence will be correlated or anticorrelated with UP states. Though strong feedforward excitation in CA3 reproduces the widely reported correlation of ripple activity with UP states (Sirota et al., 2003; Battaglia et al., 2004; Isomura et al., 2006; Mölle et al., 2006), our model predicts that a selective blockade of the mossy fiber input on CA3 pyramidal cells or an enhancement of UP-state interneuronal spiking will reduce the UP-state ripple occurrence and even reverse it to a DOWN-state correlation.

The modeled CA3 pyramidal population can be separated into two groups, one being driven to spike by the UP state input and the other silenced by the enhanced inhibition from neighboring interneurons. A similar distinction was also found through *in vivo* intracellular recordings on anesthetized mice, where CA3 pyramidal cell membrane potentials exhibited a mixed SO modulation, some being correlated with the UP state and some anticorrelated (Hahn et al., 2007). However, a study on anesthetized and naturally sleeping rats reported preferential firing of most cells during DOWN states, organized in gamma oscillations, while SWRs are still UP-correlated (Isomura et al., 2006). We propose that the increased DOWN-activity could be due to the gamma oscillations producing more overall firing than population bursts (Isomura et al., 2006; Sullivan et al., 2011), while population bursts, involving a cell minority, are still organized by the mossy input, giving rise to UP-state SWRs. Since the current version of our CA3 network does not support gamma frequency oscillations, this remains to be examined. Moreover, species-, anesthetic- or preparation-specific differences between the two studies could result in differences in the input that CA3 receives through the SO, thus affecting the size of the two subgroups.

Given the entrainment of the DG by the SO (Isomura et al., 2006; Hahn et al., 2007; Sullivan et al., 2011) CA3 is expected to be under a barrage of dentate input during UP states, which is largely absent in DOWN states. However, our *in vivo* data analysis suggests that the excitatory input received by CA1 during SWRs is roughly fixed, irrespective of the SO state during which it took place, implying that the CA3 population bursts yielding SWRs do not vary substantially in either state. This is suggestive of an intrinsic self-organized activity in CA3, promoted by the UP state input, but not shaped by it, similar to the independence of the hippocampal circuit organization proposed in the framework of theta oscillations (Mizuseki et al., 2009). The CA3 architecture of extensive recurrent excitation combined with feedback inhibition can support such self-organization.

### Temporoammonic and Schaffer inputs

The ability of the TA-CA1 synapse to exhibit both long-term depression and potentiation (Remondes and Schuman, 2002), and the dependence of CA1 spikes on the timing difference between the TA and the Schaffer inputs (Empson and Heinemann, 1995; Remondes and Schuman, 2002; Jarsky et al., 2005), suggest the importance of the TA input during SWS. Correlations of R-LM interneurons (Freund and Buzsáki, 1996) with the SO (Hahn et al., 2006), and of CA1 spiking with persistent entorhinal UP-state activity (Hahn et al., 2012), and deficiencies of TA-lesioned rats in memory retrieval (Remondes and Schuman, 2004; Suh et al., 2011) support an active role of TA input in promoting the SO entrainment to the hippocampus and its importance in spatial memory consolidation.

Our model suggests that strong Schaffer drive to CA1, variable for pyramidal cells, is sufficient to reproduce coherent interneuronal ripple oscillations, but the majority of pyramidal cells would be silenced during UP states (Taxidis et al., 2012), reminiscent of the UP-state hyperpolarization of CA1 cells found in anesthetized mice (Hahn et al., 2007), but contradicting the observed UP-state correlated spiking in naturally sleeping rats [Figure 6 and Isomura et al. (2006)]. This discrepancy was overcome in the model by the inclusion of the SWR-independent TA input that promoted UP-state pyramidal firing without critically influencing SWR characteristics. Our simulations suggest that factors, such as anesthesia, that may suppress the TA input or alter it in favor of interneuronal activity could yield the observations in (Hahn et al., 2007). However, a specific minority of “strongly Schaffer-driven” cells would still fire in ripples, mostly during UP states, in contrast with the rest of the population. This overall suppression of UP-state spiking in most cells, particularly during UP-state ripples, may underlie the temporal association memory deficits that were recently observed in transgenic mice where entorhinal cells were inhibited, depriving CA1 cells from the TA input (Suh et al., 2011).

Our combined modeling and *in vivo* analysis indicates that the main factor for determining the overall correlation of a CA1 pyramidal cell with either of the SO states is the TA input. Since most cells fire mostly during UP states, we propose that this input is largely excitatory for most of the CA1 population. Conversely, the main factor for determining whether a cell will actively participate in ripple spiking is the Schaffer input, which appears to vary substantially among CA1 cells. Our observed variability in the firing responses to SWRs has also been reported elsewhere, for both CA1 pyramidal cells (Royer et al., 2012) and interneurons (Csicsvari et al., 2000). Although in the model the variability is implemented in the number of Schaffer connections pyramidal cells receive, it may also be linked to either the strength of the Schaffer input on different CA1 pyramidal cells (Fernández-Ruiz et al., 2012) or in the convergence of Schaffer collaterals on CA1 cells or assemblies (Takahashi et al., 2012).

Apart from the effective segregation of the pyramidal population according to which SO state each cell correlates with and whether it actively participates in ripple spiking, CA1 pyramidal cells have also been shown to be segregated according to whether or not they shift their theta phase correlation between REM and non-REM sleep (Mizuseki et al., 2011). 35.1% of recorded cells that did exhibit a REM-related shift (a similar ratio to our strongly-Schaffer-driven group), also showed stronger entrainment by the SO and were more active during ripple spiking, and can be linked to our “strongly Schaffer-driven” cells. These cells are located mostly in deep sublayers of the CA1 stratum pyramidale, indicating that such sublayers may underlie different functional purposes (Mizuseki et al., 2011). Whether, these groups also reflect different functional roles, potentially linked to memory consolidation processes, remains to be examined.

### Model extensions

The relatively simple network architecture developed here, offers a first modeling approach to the problem of SO-hippocampal correlations, shedding light on the role of important parameters and key components, but omitting many features of hippocampal circuitry and cortico-hippocampal connections. Circuits such as monosynaptic projections from CA1 to the PFC, from entorhinal cortex to CA3 through the perforant path, or even commissural CA3 connections (Andersen et al., 2007) can provide delayed feedback mechanisms that may potentially have a serious effect on the timing and the excitation-to-inhibition ratio of the UP state input received by the two CA areas. Such circuits provide interesting potential extensions to the model, whose effects cannot be readily predicted.

A simplification in our model architecture is the omission of UP-state signal filtering by the DG. *In vitro* studies revealed that the mossy output to CA3 pyramidal cells can be excitatory or inhibitory, depending on the spiking frequency of the dentate cells in combination with facilitation (depression) of excitatory (inhibitory) inputs (Mori et al., 2004). Therefore, the UP state signal arriving in CA3 and the induced excitation-to-inhibition ratio could be altered by dentate activity. The future addition of a distinct DG network to our model could shed some light on the final form of the UP state signal that arrives in CA3.

SWR generation in the model is based solely on perisomatic inhibition, excluding various types of interneurons that are involved in SWRs (Klausberger and Somogyi, 2008). Recent optogenetic studies have shown dendritic interneurons to be critical for the pyramidal output (Lovett-Barron et al., 2012; Royer et al., 2012) or the regulation between TA and Schaffer inputs (Leão et al., 2012) and computational models have included a variety of interneuronal types in mechanisms for place cell pattern learning and replay (Cutsuridis and Hasselmo, 2010, 2011). Future model versions can address the role of such interneurons on the cortico-hippocampal communication and ripple generation.

The inclusion of neuronal networks from other brain areas, like the DG or thalamic subregions which influence hippocampal activity, would shed further light on the general cortico-hippocampal cross-talk during deep sleep. Recently, it was shown that decoupling such circuitries can lead to disrupted correlations between intrinsic hippocampal and thalamocortical oscillations (Phillips et al., 2012). Most importantly, the inclusion of plasticity in the involved synapses and of more complex membrane currents would also help a detailed study of the process of temporal pattern transfer through the hippocampus and eventually to PFC during SWRs, for memory consolidation.

### Conflict of interest statement

The authors declare that the research was conducted in the absence of any commercial or financial relationships that could be construed as a potential conflict of interest.
